# Icing on the Cake: “Amplification Effect” of Innovative Information Form in News Reports About COVID-19

**DOI:** 10.3389/fpsyg.2021.600523

**Published:** 2021-02-15

**Authors:** Fangfang Wen, Hanxue Ye, Yang Wang, Yian Xu, Bin Zuo

**Affiliations:** ^1^Central China Normal University, Wuhan, China; ^2^New York University, New York City, NY, United States

**Keywords:** amplification effect, COVID-19, information framing, information form, news report, risk perception

## Abstract

In the information era, the instant and diversified broadcasting of the COVID-19 pandemic has played an important role in stabilizing the societal mental state and avoiding inter-group conflicts. The presentation of visual graphics was considered as an innovative information form and broadly utilized in news reports. However, its effects on the audiences' cognition and behaviors have received little empirical attention. The current study applied real-time and retrospective priming paradigms to examine the impacts of information framing (positive vs. negative) and form (plain text vs. pie chart) on individuals' risk perception (cognition), positive emotion (emotion), and willingness to help others (behavioral intention) during the outbreak and post-pandemic period in China. The results indicated the “amplification effect” of the innovative form of information in the real-time priming condition, which increased the effect of the information framing on cognition, emotion, and behavioral intention. However, in the retrospective priming condition, the amplification effect on cognition and emotion were weakened, while its effect on behavioral intention disappeared. In conclusion, the study found the “amplification effect” of innovative information forms. Further, the difference in the results in the real-time and retrospective priming paradigms suggested the constraint of the context of the “amplification effect,” and indicated the possible deviation of the retrospective paradigm in studies about disaster-related news. This study provides empirical support for how subtle changes in information presentation influence public mental and behavioral responses during a pandemic and has important implications for media psychology and social governance.

## Introduction

Since 2020, the coronavirus disease 2019 (COVID-19) pandemic has resulted in a massive global health crisis. According to data published on the website of the World Health Organization (WHO), as of November 30, 2020, there have been more than 62 million confirmed cases including ~1.46 million deaths due to COVID-19 worldwide (WHO Coronavirus Disease (COVID-19) Dashboard, 2020). While medical experts are fighting against the coronavirus, the insights from social and behavioral science can also help to align human behavior with recommendations of public health experts and promote responses to the pandemic (Van Bavel et al., 2020).

Notably, in the context of the pandemic, conspiracy theories, fake news, and misinformation proliferated soon after the outbreak and continue to spread, with harmful consequences for social stability and outbreak control. Hence, exploring how to effectively communicate factual information is of great significance for stabilizing social mentality and avoiding rumors (Gilles et al., 2013; Van Bavel et al., 2020). Moreover, it has been confirmed that the specific forms by which information is presented in the news may have noteworthy impacts on individuals' psychological state (Kaspar et al., 2016). From the perspective of media psychology, this research aimed to explore the influence of information framing and forms of information presentation on people's risk perception, emotion, and behavioral intention.

Research in the field of media psychology has confirmed that news reports and government messages, as common and important information sources in the context of social crisis, have important impacts on the mental states and behavior patterns of the public (Busselle and Shrum, 2003; Schroeder and Pennington-Gray, 2015; Holbert et al., 2017). A series of related studies during the COVID-19 outbreak has supported the significant role of appeals and messages in promoting people's response toward the pandemic (Bilancini, 2020; Capraro and Barcelo, 2020a,b; Everett et al., 2020; Jordan et al., 2020; Pfattheicher et al., 2020; Heffner et al., 2021). However, the available studies have mainly focused on the effects of appeals or persuasive messages, while overlooking the exploration of how factual information (news) can be conveyed effectively.

News requires text, pictures, or graphs as the carrier; thus, the information forms are crucial to the impacts of news (Sundar, 2000; Kaspar et al., 2016; Lee and Kim, 2016). Previous literature has indicated that text border colors and font styles can influence the audience's cognitive processing of news information (Gerend and Sias, 2009; Kaspar et al., 2015). However, these studies primarily emphasized marginal factors such as colors or marks, while ignoring the variance of the presentation forms of the news itself (Kaspar et al., 2016). With the advent of the new media era, the carrier of news has changed from newspapers to more internet-based and digitalized platforms. Adding visualized charts to news as an auxiliary tool for information presentation is an important innovation trend in data-based news (Smiciklas, 2012; Treadwell et al., 2016). This approach has been widely practiced in well-known media including the New York Times (U.S.) and the Caixin.com (China). Compared with traditional plain-text forms, graphical data news has been regarded as an innovative and trendy form of news presentation by many researchers (De Haan et al., 2018; Cairo, 2019; Liu et al., 2020). On the one hand, research has shown that this innovative graphic form of information presentation could enhance the audience's appraisal and preference for the news, especially for readers with lower information processing capacity and news-reading motivation (Michas and Berry, 2000; Chabani and Hommel, 2014; Ellahe and Hommel, 2014; Lee and Kim, 2016). On the other hand, some studies have also suggested that compared with plain text, the application of infographic presentation can lead readers to engage in greater levels of issue-relevant thinking (Lazard and Atkinson, 2015; Huang et al., 2019). However, current research on innovative information presentation forms only deals with the impacts on overall cognition or evaluation; hence, detailed exploration of its impacts on emotion, risk perception, and behavioral intention during the pandemic is of great significance. In order to reduce the interference of additional variables, the standardized pie chart was used as a representative of an innovative information form in this study, and its effect is compared with that of news in traditional plain-text form.

Information framing is another critical characteristic of news reports (Sun et al., 2016; De Hoog and Verboon, 2020). As suggested by the “framing effect,” the positive or negative manner in which the same news is presented will affect the audiences' perception of the news, resulting in different emotions, attitudes, and behavioral responses (Scheufele, 1999; Igartua et al., 2012). It has been found that in reports of disaster incidents, emphasizing positive rather than negative information, such as rescue results and people's helping behaviors during the disaster can contribute to the reduction of negative emotions and the risk perception raised by the crisis (Borah, 2009; Balzarotti and Ciceri, 2014; Lan et al., 2019).

The exploration of the effects of information forms and framing in news presentation has achieved certain results. However, to date, research has mainly focused on investigating the effects of the two variables separately, while ignoring the possible interaction between them (e.g., Kaspar et al., 2016; Lee and Kim, 2016; Sun et al., 2016). Hence, the present study aims to explore the effects of different information presentation forms under both positive and negative information framing. Previous work has suggested that innovative forms of news presentation can promote the ability and motivation of individuals to engage in elaborate information processing (Chabani and Hommel, 2014; Kaspar et al., 2016; Lee and Kim, 2016). Based on the dual-process theory and the elaboration likelihood model (ELM, Petty and Cacioppo, 1986; Evans and Stanovich, 2013), when the possibility of refined processing increases, people are more likely to use the central path to process information; that is, they will actively apply more comprehensive methods to understand information, which is more likely to cause changes in their relevant attitudes and behavior (Lee and Kim, 2016). Therefore, we hypothesize that when presenting the news in an innovative manner with visualized graphs, the positive or negative information will have a greater impact on individuals' mental state and behavioral intention, showing the “amplification effect” (H1).

In the context of disasters, emotion and risk perception are important psychological reactions that have been widely explored in studies on news communication (Van Bavel et al., 2020). In terms of emotions, evidence from a large number of studies has demonstrated the negative effects of disaster news on emotion, and indicated that exposure to news on terrorist attacks or natural disasters will increase individuals' anxiety and depression levels (Pfefferbaum et al., 2000; Kaplan, 2008; McIntyre and Gibson, 2016; Vliegenthart and Boukes, 2018; Aslam et al., 2020). Apart from the influence of news reports on emotions, Lowry et al. (2003) found a relationship between news reports and public risk perceptions. Research has shown that the public's perception of social crime rates was not based on actual crime rates, but by the prominence of crime incidents in news reports (Gerbner et al., 1986; Lowry et al., 2003; Romer et al., 2003; Boukes and Vliegenthart, 2017; Abrams and Greenhawt, 2020). However, previous studies mainly focused on the negative effects of disaster news while ignoring their possible positive contributions (e.g., Unz and Schwab, 2008; Vliegenthart and Boukes, 2018; De Hoog and Verboon, 2020). Studies have shown that news reports on disaster conditions can also stimulate the viewers' empathy for victims, and further promote their willingness and behavior to help others (Simon, 1997; Gasser and Solé, 2006). Meanwhile, according to research conducted during the outbreak of SARS, high-level risk perception also motivated people to adopt altruistic behaviors in order to reduce their perceived risks in the surrounding environment (Xie et al., 2005). Based on these facts, the current research also investigates participants' intention to help in the context of the COVID-19 pandemic, aside from measurement of their emotions and perceptual changes. We hypothesized that innovative presentation of the negative news could also amplify people's willingness to help others (H2).

Most of the existing research was conducted using the retrospective priming paradigm, which investigated the influence of news reports long after the disasters occurred and lacked real-time measurement (e.g., Marshall et al., 2007; Ben-Zur et al., 2012). However, it is possible that retrospective studies may be biased because of the constructive nature of memory and time-varying factors such as controllability of disaster and personal-disaster relevance, which exert influences on people's retrospective memory (Balzarotti and Ciceri, 2014; Boukes and Vliegenthart, 2017). Therefore, it is necessary to combine and compare the retrospective and real-time approaches in exploring the influence of information forms and framing on public risk perception, emotions, and willingness to help, specifically in the context of the global COVID-19 pandemic. Based on previous explorations, we hypothesized that the influence of information forms and framing are more prominent when using the real-time priming paradigm, and that they will be less stable when using the retrospective priming paradigm (H3).

Taken together, by conducting two experiments, the current research combined the real-time and retrospective priming paradigms to investigate the influence of information forms and framing on people's risk perception, emotion, and willingness to help during the COVID-19 pandemic in mainland China. This research aims to provide preliminary evidence of the “amplification effect” of innovatively presented news forms. It could promote the understanding of the psychological effects of news media and provide implications on how to present disaster information in a better way, so as to encourage the public's response to the pandemic.

## Experiment 1: Test Based on the Real-Time Priming Paradigm

This experiment applied the real-time priming paradigm to present the pandemic situation news. The purpose of this study was to explore the effects of the framing and forms of news information on viewer's risk perception, positive emotion, and willingness to help during the COVID-19 pandemic situation, and provide a preliminary examination of the “amplification effect” of the innovative information form.

### Method

#### Participants

According to G^*^Power (Faul et al., 2009), we would need 195 participants for each task to attain an 80% chance of detecting an effect of 0.3. As such, a total of 252 participants were recruited through an online platform, of which 73 were male and 179 were female. The mean age of the sample was 27.46, ranging from 18 to 66. All participants enrolled in the study voluntarily, with informed consent prior to its initiation.

#### Design and Measures

A 2 (information framing: positive vs. negative) × 2 (information form: plain text vs. pie chart) between-subject design was applied in the study. Among these, real-time data of people cured during the pandemic were presented as positive valance information, while data on death was presented as negative valance information. In addition, the information was presented in the form of plain text under the traditional condition, while visual graphics were added in the innovative form. The dependent variables included the severity dimension of risk perception, positive emotion, and willingness to help others.

The severity dimension of risk perception was measured using the “Risk Perception Questionnaire” compiled by Xie et al. (2005) in their research on SARS, which includes the dimensions of “the possibility of infection,” “severity,” “uncertainty,” and “uncontrollability.” These dimensions are the risk characteristics of SARS obtained by Xie et al. in the later stage of the 2003 SARS epidemic in China (May 2003) by investigating the impact of the SARS epidemic crisis on the mental state of the public through questionnaires. This instrument was also used to measure the public's risk perception in the COVID-19 pandemic (Wen et al., 2020). The current study mainly focuses on the impact on the “severity” dimension of risk perception. The item that measures the severity perception of COVID-19 pandemic was used, and the term “SARS” in the original item was modified to “coronavirus disease:” “The coronavirus disease is most likely to cause death.” This item was rated on a 5-point scale ranging from 1 (*totally disagree*) to 5 (*totally agree*).

Since the concern of this study is the impact on the individual's positive feelings, we selected two items of “pleasure” and “safety” to represent the main positive emotions during the pandemic, and organized the items with reference to the narrative in the classic State-Trait Anxiety Inventory-Form (STAI) (Spielberger and Gorsuch, 1983): “I feel pleasant” and “I feel secure.” The participants were requested to evaluate their feelings on a 4-point scale ranging from 1 (*not at all*) to 4 (*very much so*). In this study, Cronbach's alpha was 0.62. The sum of the scores of the two items was defined as the final score of positive emotion.

Willingness to help was measured using two self-compiled items. The current study was conducted in the early stage of the COVID-19 pandemic in China (January 24 to February 9, 2020). The unclear information on disease protection and the shortage of masks were the prominent problems during that period. Combining these social problems with common prosocial behaviors in news reports, we selected two of the most representative helping behaviors: “I am willing to teach others how to prevent the coronavirus disease” and “If I go to a pharmacy to buy masks and find that there are few left, I will leave some masks for the next person.” These items were rated on a 5-point scale ranging from 1 (*totally disagree*) to 5 (*totally agree*). The sum of the scores of the two items was defined as the final score of willingness to help.

#### Procedure

**Figure 1:**
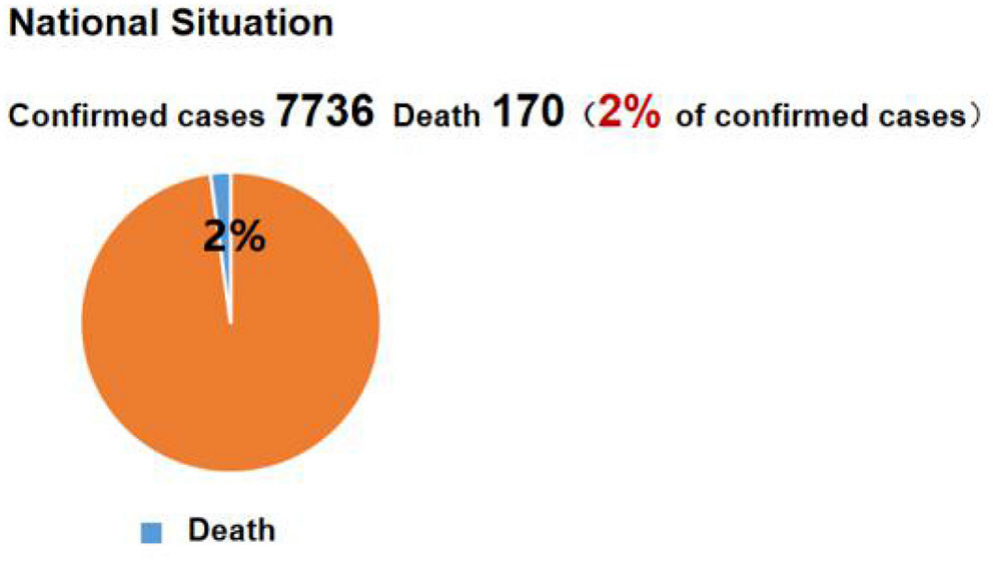
The illustration of innovative priming information.

**Figure 2:**
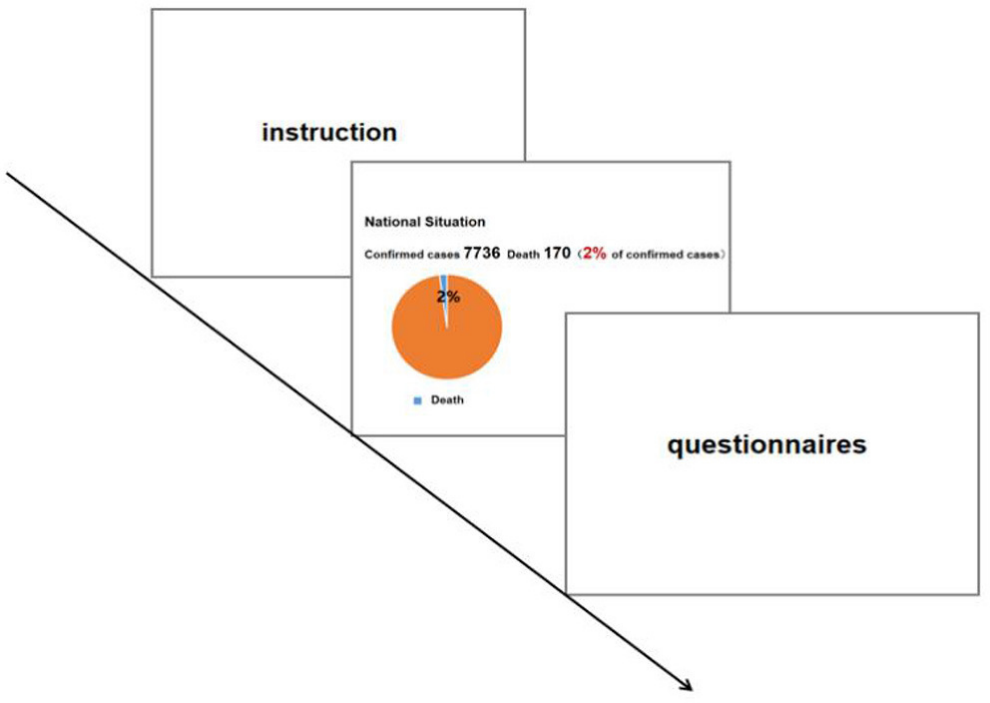
The procedure of Experiment 1 and Experiment 2.

This experiment was conducted in the early stage of the COVID-19 outbreak in China (i.e., from January 24 to February 9). Since the laboratory where we were supposed to conduct the study was not available during the pandemic, this experiment was conducted using an online survey platform (www.wjx.com). Participants were randomly assigned to four conditions, and each condition was primed with either positive or negative news related to the domestic pandemic situation in either plain text or pie chart form (Figure 1). The news was taken from real-time reports on the domestic pandemic situation from an online news platform (www.people.com). As of January 30, 2020, the number of confirmed COVID-19 cases in China was 7,736, of which 124 had recovered (2% cure rate), and 170 had died (2% mortality rate). In the negative-plain text condition (*n* = 63), participants were required to read news reports about deaths presented in pure text form; in the negative-pie chart condition (*n* = 63), a visual pie chart was added to demonstrate the mortality rate. Conversely, in the positive-plain text condition (*n* = 63), participants were required to read news about the number of people cured of COVID-19, which was presented in pure text form; in the positive-pie chart condition (*n* = 63), a visual pie chart was added to demonstrate the recovery rate. The participants were required to carefully read the news within 15 s. After the priming stage, participants were then required to evaluate their risk perception (the severity dimension), positive emotion, and willingness to help, and finally filled in their demographic characteristics, including gender and age (Figure 2).

### Results

#### Descriptive Statistics

SPSS 23.0 was used to analyze the data. The outcome of the descriptive statistics is displayed below (see Table 1).

**Table 1:** Descriptive statistics of risk perception, positive emotion, and willingness to help of participants in different information priming conditions (*M* ± *SD*).

**Information framing**	**Information form**	**Severity dimension of** **risk perception**	**Positive emotion**	**Willingness to help**
Negative	Pie chart	3.67 ± 1.18	4.38 ± 1.54	8.29 ± 1.43
	Plain-text	3.48 ± 1.08	5.23 ± 1.58	7.65 ± 1.79
Positive	Pie chart	3.17 ± 1.04	4.52 ± 1.28	8.27 ± 1.59
	Plain-text	3.65 ± 1.05	4.65 ± 1.45	8.48 ± 1.48

#### Severity Dimension of Risk Perception

**Figure 3:**
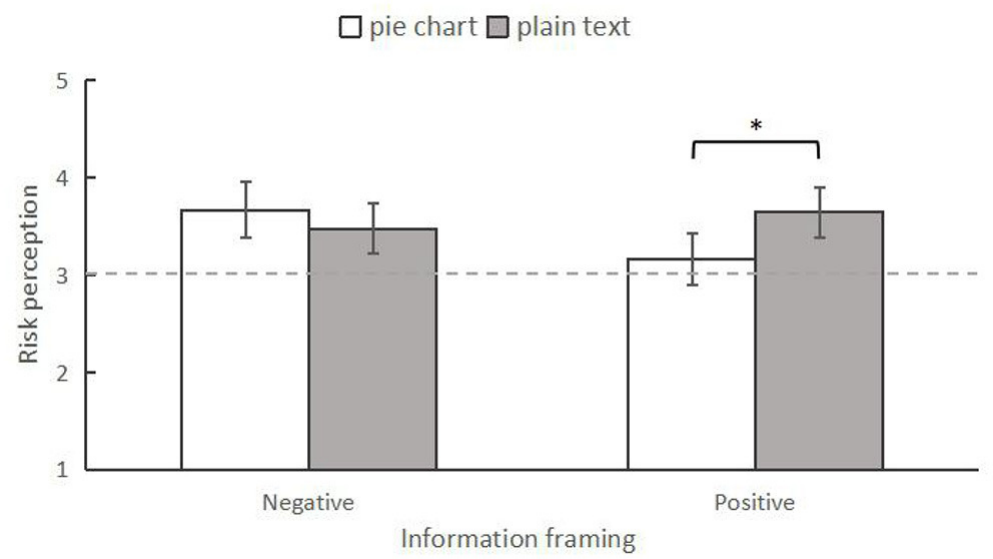
The interaction between information frame and information form on risk perception in experiment 1. *p < 0.05. The error bars indicated 95% Confidence Intervals (95% CI).

First, a 2 (information framing: positive vs. negative) × 2 (information form: plain text vs. pie chart) between-subjects two-way ANOVA was conducted to analyze the differences in risk perception among the four conditions. The results indicated that there was no significant main effect for information framing, *F*_(1,246)_ = 2.377, *p* = 0.124, $\eta_{\text{p}}^{2}$ = 0.010. The main effect of information form was also not significant, *F*_(1,246)_ = 1.611, *p* = 0.206, $\eta_{\text{p}}^{2}$ = 0.007. However, the interaction effect between them was significant, *F*_(1,246)_ = 4.884, *p* = 0.028, $\eta_{\text{p}}^{2}$ = 0.019 (Figure 3). Specifically, using simple effect analysis, we found that when presenting positive news, individuals who read the information in the pie chart form (*M* = 3.13, *SD* = 0.14) had lower perceived severity than those who read it in the plain text form (*M* = 3.63, *SD* = 0.14), *F*_(1,246)_ = 6.379, *p* = 0.012, $\eta_{\text{p}}^{2}$ = 0.025. However, respondents in the negative news condition did not show any difference in risk perception for the two forms of information presentation (pie chart form: *M* = 3.67, *SD* = 0.14; plain text form: *M* = 3.54, *SD* = 0.14), *F*_(1,246)_ = 0.351, *p* = 0.554, $\eta_{\text{p}}^{2}$ = 0.001.

#### Positive Emotion

**Figure 4:**
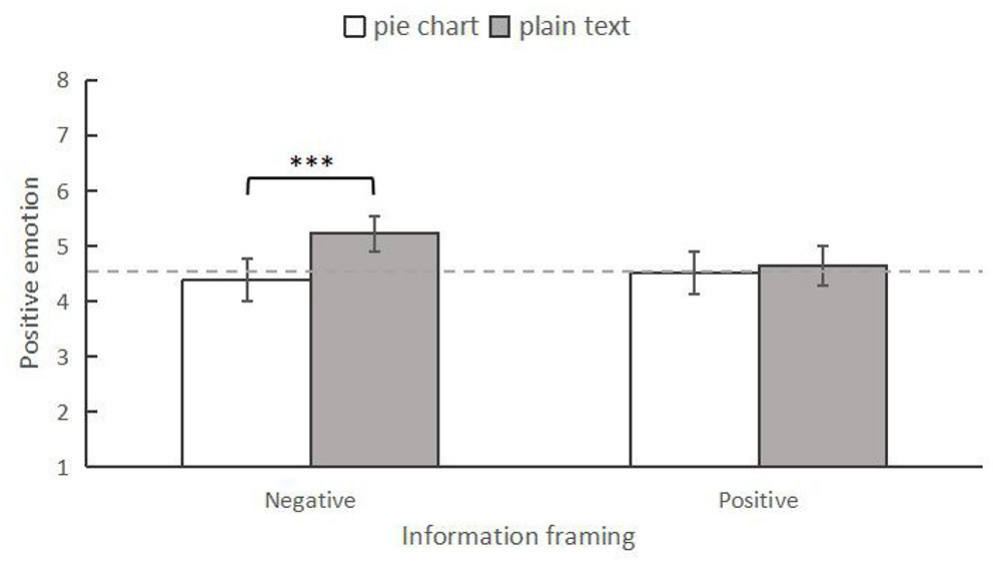
The interaction between information frame and information form on positive emotion in Experiment 1. ***p < 0.001. The error bars indicated 95% CI.

Similarly, a two-way ANOVA was conducted to examine the differences in positive emotion among the four conditions. The results indicated that the main effect of information form was significant, *F*_(1,246)_ = 9.548, *p* = 0.002, $\eta_{\text{p}}^{2}$ = 0.037, while the main effect of information framing was not significant, *F*_(1,246)_ = 1.535, *p* = 0.216, $\eta_{\text{p}}^{2}$ = 0.006. In addition, the interaction effect of the two variables was significant, *F*_(1,246)_ = 5.678, *p* = 0.018, $\eta_{\text{p}}^{2}$ = 0.023 (Figure 4). Specifically, using simple effect analysis, we found that when presenting negative news, individuals who read the information in the pie chart form (*M* = 4.30, *SD* = 0.19) had lower positive emotion than those who read it in the plain text form (*M* = 5.33, *SD* = 0.19), *F*_(1,246)_ = 13.469, *p* < 0.001, $\eta_{\text{p}}^{2}$ = 0.052. However, those in the positive news condition did not show any difference in positive emotion (pie chart form: *M* = 4.49, *SD* = 0.19; plain text form: *M* = 3.67, *SD* = 0.19), *F*_(1,246)_ = 0.495, *p* = 0.482, $\eta_{\text{p}}^{2}$ = 0.002.

#### Willingness to Help

**Figure 5:**
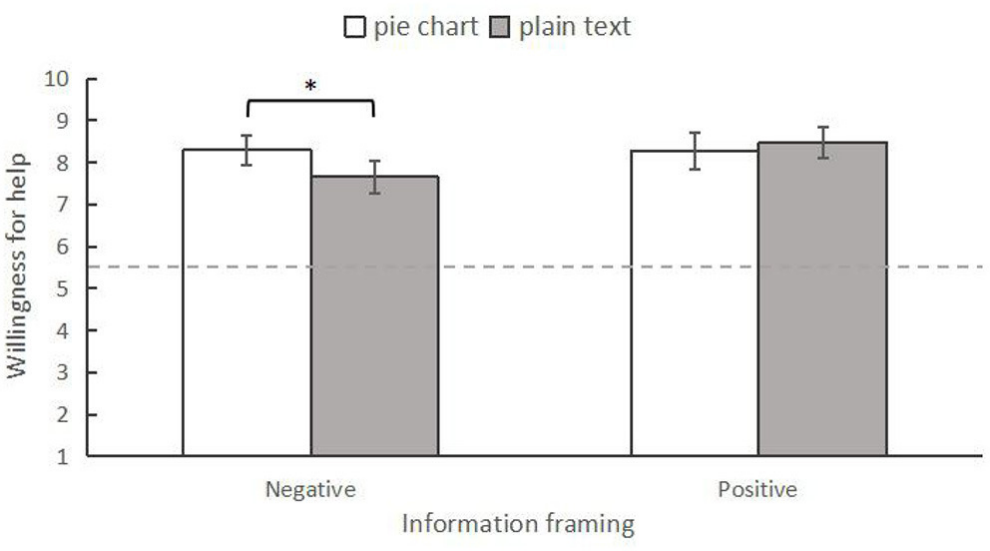
The interaction between information frame and information form on willingness to help in Experiment 1. * *p* < 0.05 The error bars indicated 95% CI.

Similarly, a two-way ANOVA was conducted to examine the differences in willingness to help among the four conditions. The results indicated that there was no significant main effect for information framing, *F*_(1,246)_ = 2.615, *p* = 0.107, $\eta_{\text{p}}^{2}$ = 0.011. The main effect of information form was also not significant, *F*_(1,246)_ = 1.228, *p* = 0.269, $\eta_{\text{p}}^{2}$ = 0.005. However, the interaction effect between them was significant, *F*_(1,246)_ = 4.413, *p* = 0.037, $\eta_{\text{p}}^{2}$ = 0.018 (Figure 5). Specifically, using simple effect analysis, we found that when presenting negative news, individuals who read the information in the pie chart form (*M* = 8.33, *SD* = 0.20) had a higher willingness to help than those who read it in plain text form (*M* = 7.67, *SD* = 0.21), *F*_(1,246)_ = 4.775, *p* = 0.030, $\eta_{\text{p}}^{2}$ = 0.019. However, those in the positive news condition did not show any difference in willingness to help by information form (pie chart form: *M* = 8.25, *SD* = 0.20; plain text form *M* = 8.44, *SD* = 0.20), *F*_(1,246)_ = 0.454, *p* = 0.501, $\eta_{\text{p}}^{2}$ = 0.002.

### Discussion

Based on the real-time priming paradigm, the first experiment preliminarily explored the effects of information framing and form in pandemic-related news on individuals' risk perception, positive emotion, and willingness to help others during the COVID-19 crisis. In general, the results suggest that information valance and form in the news have important influences on the severity dimension of risk perception, positive emotion, and willingness to help. Furthermore, the innovative pie chart in news presentation can amplify the influence of information framing, showing an “amplification effect.” Specifically, when presenting positive news, the innovative form with visual graphics can enhance the effect of a positive framing, and help to reduce participants' risk perception of the severity of the pandemic. However, when presenting negative news, the application of innovative information can enhance the effect of negative framing, thereby reducing positive emotions, but at the same time, improving their willingness to help others.

In conclusion, the results of Experiment 1 confirmed the “amplification effect” of innovative information form; it makes positive information more positive, and the negative information more negative. However, this study was conducted during the serious outbreak of COVID-19, raising the question of whether the amplification effect of innovative information form still exists after this period. Hence, the second experiment will use the retrospective priming paradigm to further explore this question.

## Experiment 2: Test Based on a Retrospective Priming Paradigm

Experiment 2 used the retrospective priming paradigm to reexamine the effects of information framing and form of pandemic-related news on viewers' severity dimension of risk perception, positive emotion, and willingness to help in the context of a post-pandemic situation, in order to explore the background condition of the “amplification effect” of the innovative information form.

### Method

#### Participants

According to G^*^Power (Faul et al., 2009), 195 participants would be required for each task to attain an 80% chance of detecting an effect of 0.3. Thus, a total of 200 participants were recruited through an online platform. After excluding the data of participants who had responded too quickly or had missing data, the final sample was 187, of which 48 were male and 139 were female. The mean age of the sample was 23.71, ranging from 18 to 44. All participants enrolled in the study voluntarily, with informed consent prior to its initiation.

#### Design and Measures

This experiment also applied a 2 (information framing: positive vs. negative) × 2 (information form: plain text vs. pie chart) between-subject design. The dependent variables were the participants' severity dimension of risk perception, positive emotion, and willingness to help after reading the priming material. The materials and measurements were the same as in Experiment 1.

#### Procedure

The procedure of Experiment 2 was similar to that of Experiment 1; however, it was carried out during the post-pandemic period in China. Since the laboratory where we were supposed to conduct the study was not available during the pandemic, this experiment was conducted using an online survey platform (www.wjx.com). This experiment adopted the retrospective priming paradigm; thus, it started by introducing participants with a guide question: “Imagine you are back in January 2020, and the next page is a news report about the national pandemic situation at that time. Please carefully read the information and answer the following questions.” The 187 participants were randomly assigned to one of four experimental conditions and then presented with positive or negative pandemic-related news in either plain text or pie chart forms. After 15 s of reading, the participants were requested to evaluate their risk perception, positive emotion, and willingness to help, and finally provided their demographic information, such as gender and age.

### Results

#### Descriptive Statistics

SPSS 23.0 was used to analyze the data. The outcome of the descriptive statistics is displayed below (see Table 2).

**Table 2:** Descriptive statistics of risk perception, positive emotion, and willingness to help of participants in different information priming conditions (*M* ± *SD*).

**Information framing**	**Information form**	**Severity dimension of** **risk perception**	**Positive emotion**	**Willingness to help**
Negative	Pie chart	3.28 ± 1.10	4.02 ± 1.26	7.98 ± 1.67
	Plain-text	3.74 ± 0.92	3.79 ± 1.21	8.13 ± 1.54
Positive	Pie chart	3.75 ± 1.08	3.77 ± 1.43	7.93 ± 1.61
	Plain-text	3.66 ± 0.98	4.47 ± 1.89	7.94 ± 1.55

#### Severity Dimension of Risk Perception

**Figure 6:**
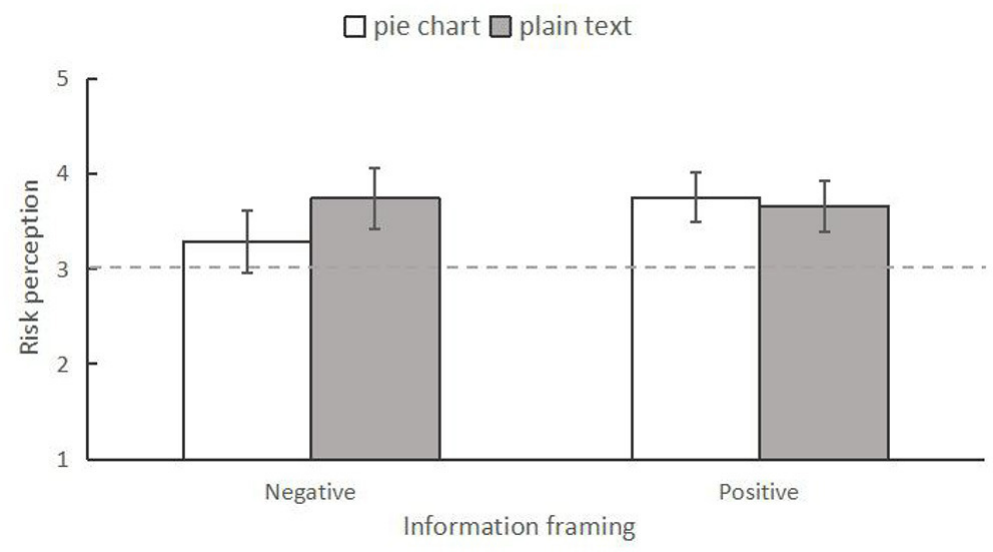
The interaction between information frame and information form on risk perception in Experiment 2. The error bars indicated 95% CI.

As in Experiment 1, a 2 (information framing: positive vs. negative) × 2 (information form: plain text vs. pie chart) between-subjects ANOVA was conducted to analyze the differences in risk perception among the four conditions. The results indicated that there was no significant main effect for information framing, *F*_(1,181)_ = 1.843, *p* = 0.176, $\eta_{\text{p}}^{2}$ = 0.010. The main effect of information form was also not significant, *F*_(1,181)_ = 1.595, *p* = 0.208, $\eta_{\text{p}}^{2}$ = 0.009. However, the interaction effect between them was marginally significant, *F*_(1,181)_ = 2.990, *p* = 0.086, $\eta_{\text{p}}^{2}$ = 0.016 (Figure 6). Specifically, using simple effect analysis, we found that when presenting negative news, individuals who read the information in the pie chart form (*M* = 3.27, *SD* = 0.16) had lower perceived severity than those who read it in the plain text form (*M* = 3.73, *SD* = 0.16), *F*_(1,181)_ = 4.472, *p* = 0.036, $\eta_{\text{p}}^{2}$ = 0.024. However, those in the positive news condition did not show any difference in risk perception (pie chart form: *M* = 3.75, *SD* = 0.16; plain text form: *M* = 3.68, *SD* = 0.15), *F*_(1,181)_ = 0.094, *p* = 0.759, $\eta_{\text{p}}^{2}$ = 0.001.

#### Positive Emotion

**Figure 7:**
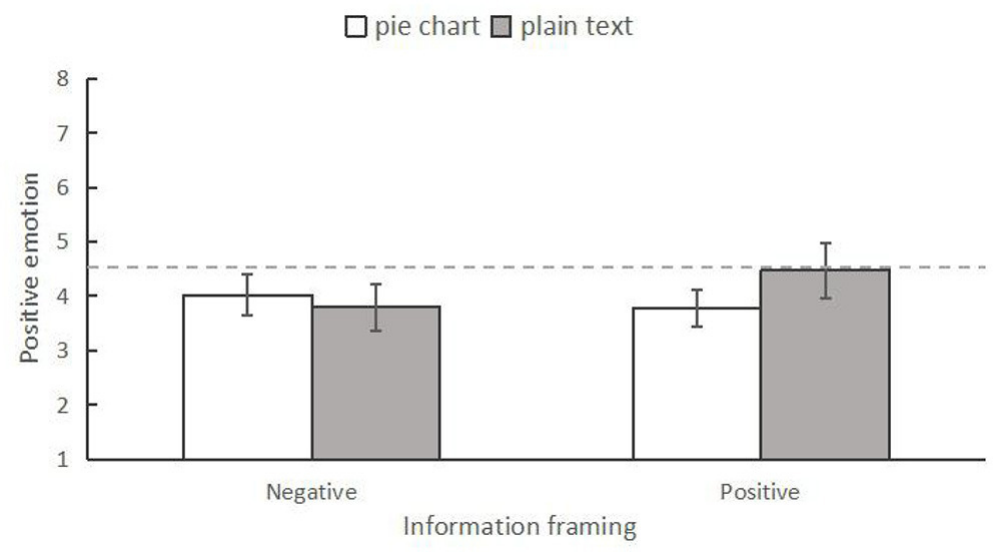
The interaction between information frame and information form on positive emotion in Experiment 2. The error bars indicated 95% CI.

Similarly, a two-way ANOVA was conducted to examine the differences in positive emotion among the four conditions. The results indicated that there was no significant main effect for information framing, *F*_(1,181)_ = 0.508, *p* = 0.477, $\eta_{\text{p}}^{2}$ = 0.003. The main effect of information form was also not significant, *F*_(1,181)_ = 0.543, *p* = 0.462, $\eta_{\text{p}}^{2}$ = 0.003. However, the interaction effect between them was marginally significant, *F*_(1,181)_ = 3.586, *p* = 0.060, $\eta_{\text{p}}^{2}$ = 0.019 (Figure 7). Specifically, using simple effect analysis, we found that when presenting positive news, individuals who read the information in the pie chart form (*M* = 03.80, *SD* = 0.23) had lower positive emotion than those who read it in the plain text form (*M* = 4.34, *SD* = 0.22), *F*_(1,181)_ = 3.426, *p* = 0.066, $\eta_{\text{p}}^{2}$ = 0.019. However, those in the positive news condition did not show any difference in positive emotions (pie chart form: *M* = 4.07, *SD* = 0.23; plain text form: *M* = 3.81, *SD* = 0.22), *F*_(1,181)_ = 0.643, *p* = 0.424, $\eta_{\text{p}}^{2}$ = 0.004.

#### Willingness to Help

**Figure 8:**
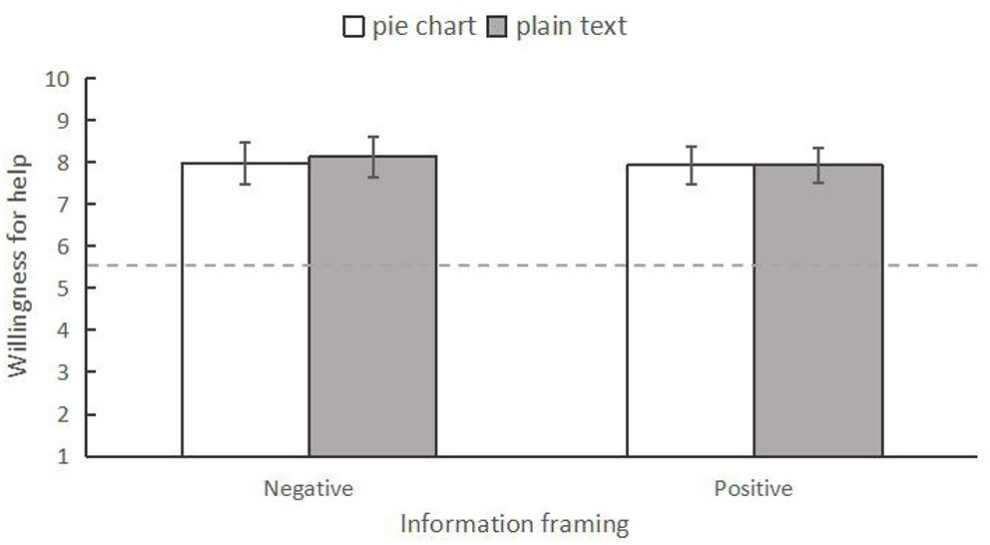
The interaction between information frame and information form on willingness to help in Experiment 2. The error bars indicated 95% CI.

Similarly, a two-way ANOVA was conducted to examine the differences in willingness to help among the four conditions. However, no significant differences were found among them (Figure 8), *F* < 0.60, *p* > 0.05, $\eta_{\text{p}}^{2}$ < 0.01.

### Discussion

In Experiment 2, based on the retrospective priming paradigm, the effects of news information framing and form on risk perception, emotion, and willingness to help were explored under the post-pandemic situation in China, when the coronavirus disease is less concerning. In general, the results of this study were different from those of Experiment 1. The effects of information framing and information form on individuals' risk perception and positive emotion were weakened, and the effects on willingness to help disappeared. Furthermore, the influential tendency of information form is opposite to the “amplification effect” in the first study.

In particular, when negative news was presented, a pie chart form led to lower risk perception. On the other hand, when positive news was present, a pie chart form led to lower positive emotion. The weakening of the two-variable effects of information framing and form may be due to participants' reduced personal relevance and increased coping potential to the pandemic (Balzarotti and Ciceri, 2014). Nevertheless, the opposite trend of the “amplification effect” will be further discussed in the section below.

## General Discussion

This research conducted two experiments to examine the impacts of innovative information forms and information framing on individuals' risk perception, positive emotions, and willingness to help in the periods of high-risk pandemic and normalized pandemic prevention. In general, both experiments demonstrated the impact of information forms and framing on individuals' psychological states and behavioral intentions, although the patterns of results were not completely consistent between the experiments. These results indicated that apart from the increase in preference for news found in a previous study (Lee and Kim, 2016), the innovative presentation of graphic information is beneficial to individuals' elaborate processing of news, which leads to greater cognitive or behavioral changes. The influence of information framing shown in this research such as the negative association between positive information and risk cognition in Experiment 1, demonstrated the existence of a framing effect in news reports and its potential influence on individuals' cognitive processes (Borah, 2009; Balzarotti and Ciceri, 2014).

More importantly, the “amplification effect” was clearly established by the significant interaction between information forms and framing on participants' risk cognition, positive emotion, and willingness to help in the first experiment. When presenting the news with positive framing, that is, information regarding the number of cured people, innovatively adding visualized graphics amplified the positive effect of the information and significantly reduced the severity dimension of risk perception. On the other hand, when presenting news with negative framing, that is, information regarding the number of deaths, adopting the innovative forms of information presentation significantly reduced the positive emotions of participants while promoting their willingness to help others. In brief, the innovative way of presenting information “amplifies” the influence of positive and negative information framing on the individual's mental state and behavioral intention. This finding is consistent with the results of previous studies, which indicated that the elaboration, acquisition, and evaluation of news could be enhanced by infographic information (Lazard and Atkinson, 2015; Lee and Kim, 2016). Meanwhile, it has also been found that, compared with plain text, the application of infographic presentation will encourage readers to engage in greater levels of issue-relevant thinking (Lazard and Atkinson, 2015; Lee and Kim, 2016; Huang et al., 2019). The current study further extends this amplification effect to the field of disaster data-based news. From the perspective of cognitive processing, the “amplification effect” in the present study could be due to the increase of individuals' motivation and ability to elaborately process the data that is presented in forms of visualized graphics (Lee and Kim, 2016). According to the dual-process theory and the elaboration likelihood model (Petty and Cacioppo, 1986; Evans and Stanovich, 2013), the central and peripheral paths of information processing coexist. When the condition is more conducive to elaborate processing, the individual is more likely to process the information through the central path, which makes their attitude and behaviors more likely to be affected by the contents of the information (Petty and Cacioppo, 1986; Chabani and Hommel, 2014). Hence, compared to the text-only forms, the addition of innovative news presentation forms, such as pie charts, can lead to a more elaborate information processing procedure, and further amplify the effect of information framing on individuals' risk cognition, emotion, and willingness to help.

Experiment 2 tried to examine the results of Experiment 1 with the retrospective priming paradigm conducted during the period of normalized COVID-19 prevention in China. However, there are important differences in the interactive patterns between information forms and framing between results under two priming paradigms. To better compare the influences of the real-time and retrospective priming paradigm, a three-way ANOVA is conducted as a supplemental analysis (see in the supplemental material). Firstly, the independent-sample *T*-test and non-parametric test were performed to compare the demographic characteristics of two samples, including gender, age, educational background, social-economic status (SES), and occupation. The results showed that the samples in the two experiments only significantly differed in age and educational background. Hence, the two variables were further taken as covariates in the three-way ANOVA test (priming paradigm × information framing × information form). The results indicated that, in the analysis of positive emotion and risk perception, the effects of age and educational background were not significant, and the three-way interaction was significant. Therefore, the comparison between Experiments 1 and 2 on emotion and risk perception is meaningful. However, in the evaluation of willingness to help, there is a significant effect of education background in the dummy variable represented by graduate students. Thus, we should be more conservative for the different results of willingness to help between two experiments, which could be the by-product of education background, but it should be noted that this effect has no influence on the tendency of the amplification effect we found, confirming that the original result is robust.

Generally, the contrary impacts on positive emotion and risk perception in the two experiments may be caused by the differences in the public's perception of the personal relevance of the pandemic as well as their ability to respond to the pandemic in the two periods (Balzarotti and Ciceri, 2014). Compared with the period of normalized COVID-19 prevention, individuals will perceive a higher possibility of their own infection, and lower controllability and response potential toward the pandemic during the risk period (Xie et al., 2005). Hence, during the risk period, individuals are more likely to pay attention to relevant news, and they are more susceptible to the form and frame of the news. In addition, in order to control the influence of information content, the news information presented in Experiment 2 was consistent with that used in Experiment 1; thus, the recovery rate (January 2020, 2%) used in the materials was lower than the death (5.2%) and recovery rates in September 2020 (93.6%) [WHO Coronavirus Disease (COVID-19) Dashboard, 2020]. The positive and negative framing information in Experiment 2 may lead to the opposite effects due to the comparison with the current situation. This difference also indicates that retrospective priming research conducted after the disaster may cause deviations in the results because of the changes in individual perceptions and situations, among other reasons.

This research has important theoretical and practical contributions. At the theoretical level, this study supplemented the lack of research examining the interaction between information forms and framing in disaster news studies, and proposed the “amplification effect” of innovative information provision on information framing. In addition, previous studies related to the influence of disaster news have mainly focused on the negative effects (e.g., Vliegenthart and Boukes, 2018; De Hoog and Verboon, 2020). This research, however, simultaneously examined the positive and negative sides of disaster news reporting and suggested a way to promote the public's altruistic behavior by reporting the disaster news in “appropriate” ways. At the methodological level, this study innovatively combined the real-time and retrospective priming paradigms and revealed the difference between them. This result questions the accuracy of retrospective research and cautions researchers in applying their results. At a practical level, this research demonstrated the influence of information forms and framing on individuals' risk cognition, emotion, and willingness to help. This suggests that the government could utilize the broadcast of disaster news to stabilize social mentality and promote altruistic behavior.

In discussing the findings, we finally want to acknowledge the limitations of the current study and point to future directions. First, in order to reduce the impacts of irrelevant factors, this study simplifies the information form into two categories: traditional and innovative; and uses the widely-applied pie chart as the representative of innovative form. However, during the COVID-19 pandemic, innovative news forms including interactive infographic news and news based on short videos, for instance, Tik Tok, have also played significant roles in news communication. Therefore, how the amplification effect found in this research will change in more innovative news forms remains to be explored. Future research can further verify the amplification effect in wider news forms, referring to the updated innovation trend of news (Lee and Kim, 2016). Second, the present study only measures the difference in individuals' self-report intention of helping others after reading differently presented news. According to the suggestion of Bilancini (2020), the change of actual behavior is more costly than that of behavioral intentions, which requires stronger and more effective interventions. Hence, it remains to be verified whether the amplification effect of innovative news in our study can lead to changes in actual altruistic behavior. Finally, positive emotion, risk perception, and willingness to help are taken as three parallel dependent variables in this research. However, we did not construct a model to explain the influencing mechanism between the variables. Therefore, it is an important direction to explore the influencing mechanism of news presentation on individuals' emotion, cognition, and behavioral intention in future exploration.

## Conclusion

The current study applied the real-time and retrospective priming paradigms to examine the effects of the information framing and form on pandemic-related news during the outbreak and post-pandemic period in China. In the real-time condition, the “amplification effect” of innovative form on the power of information framing was found. Furthermore, by comparing the results of the two paradigms, the results revealed the necessary background condition of the “amplification effect.” In addition, it suggested the possible deviation of the retrospective paradigm in studies about disaster news. At the theoretical level, the current study discovered the “amplification effect” of innovative news forms for the first time. In addition, it has important implications in the choice of paradigms for relative study in the future. Practically, the findings of this study provide empirical support for the adjustment of public mental and behavioral conditions through changes in news reports during social crises.

## Data Availability Statement

The raw data supporting the conclusions of this article will be made available by the authors, without undue reservation.

## Ethics Statement

The studies involving human participants were reviewed and approved by the Ethics Committee of the Center for Studies of Social Psychology at Central China Normal University (CSSP-2020016). The patients/participants provided their written informed consent to participate in this study.

## Author Contributions

FW, BZ, and YX contributed to the design of the study. HY and YW organized, analyzed the database and wrote the different sections of the manuscript. All authors contributed to manuscript reversion, read and approved the submitted version.

## Conflict of Interest

The authors declare that the research was conducted in the absence of any commercial or financial relationships that could be construed as a potential conflict of interest.
