# Supplementary material for: Icing on the Cake: “Amplification Effect” of Innovative Information Form in News Reports About COVID-19
Source: Front Psychol. 2021 Feb 15;12:600523. doi: 10.3389/fpsyg.2021.600523 (PMC7917205; doi:10.3389/fpsyg.2021.600523)
Supplement: Supplementary file 1 [file Presentation_1.pdf]

Appendix

To better illustrate the influence of the priming paradigm, that is, the real-time and retrospective priming paradigm, a three-way ANOVA was conducted as a supplemental analysis. The priming paradigm, information frame (positive vs. negative), and information form (traditional vs. innovative) were between-participant independent variables, and the dependent variables were risk perception, positive emotion, and willingness for help.

Before formal analysis, considering the period between two waves of testing (Experiment 1 and Experiment 2), we preliminary compared the participants' demographic information. The details of participants' gender, age, self-evaluated social economic status, occupations, and education backgrounds were listed in Table 1.

Table A1. The detailed demographic information of participants in Experiment 1 and Experiment 2.

	Experiment 1		Experiment 2	
Demographic information	*M*	*SD*	*M*	*SD*
Continuous variables				
Age	27.46	0.67	23.72	0.34

Social economic status		5.05	1.58	5.16	1.56
Categorical variables		*N*	%	*N*	%
Gender	Male	73	29.0	48	25.7
	Female	179	71.0	139	74.3
Occupation	College students	157	62.3	116	62.0
	Workers	17	6.7	4	2.1
	Salesmen	13	5.2	4	2.1
	Marketing workers	6	2.4	0	0
	Custom services	9	3.6	0	0
	Administration	4	1.6	8	4.3
	Human resources	1	0.4	4	0.5
	Accounts	2	0.8	5	2.7
	Clerical staff	3	1.2	3	1.6
	Technician	2	0.8	8	4.3

	Manager	5	2.0	0	0
	Teacher	3	1.2	18	9.6
	Consultant	2	0.8	0	0
	Specialist	2	0.8	3	1.6
	Others	26	10.3	17	9.1
Educational background	Elementary school	5	2.0	0	0
	Junior high school	24	9.5	1	0.5
	High school	17	6.7	4	2.1
	Junior college	22	8.7	12	6.4
	Undergraduate	138	54.8	120	64.2
	Graduate student and above	46	18.3	50	26.7

The independent sample t-test compared participants' difference in age and self-perceived social economic status between Experiment 1 and Experiment 2. The

results only showed the significant difference on age, $t(437) = 4.54$, $p < .001$, Cohen's $d = 0.44$, while the difference on social economic status was not significant, $p = .153$. The Mann-Whitney U test was conducted for participants' differences in gender, occupation, and education background. It revealed that only education background was significantly different between two experiments, $Z = -4.45$, $p < .001$, and the effect of gender and occupation were not significant, $p_s > .44$.

Therefore, to examine the effect of the priming paradigm on participants' risk perception, positive emotion, and willingness for help, participants' age and education background were included as covariates in the three-way ANOVA (priming paradigm × information frame × information form). Considering the education background was the categorical variable with 6 levels, it was transformed into 5 dummy variables while the undergraduate level was labeled as the baseline.

Finally, the three-way ANOVA was conducted to analyze the effect of priming paradigm on risk perception, positive emotion, and willingness for help, respectively. The results indicated that, in the analysis of positive emotion and risk perception, the effects of age and educational background were not significant in all analyses, $p_s > .060$. More importantly, these analysis found the significant three-way interactions of priming paradigm, information frame, and information form in participants' evaluation of risk perception, $F(1, 425) = 7.620$, $p = .006$, $\eta_p^2 = .018$, and positive emotion, $F(1, 425) = 9.583$, $p = .002$, $\eta_p^2 = .022$. Therefore, it was meaningful to

compare their different results between Experiment 1 and Experiment 2 in terms of risk perception and positive emotion.

However, in the evaluation of willingness for help, there was a significant effect of education background in the dummy variable represented graduate students, $F(1, 425) = 7.412$, $p = .007$, $\eta_p^2 = .017$. Besides, the three-way interaction of priming effect, information frame and information form was not significant, $F(1, 425) = 1.553$, $p = .213$, $\eta_p^2 = .004$. Thus, we should be more conservative for the different result of willingness for help between two experiments, which could be the by-product of education background. But it should be noted that this effect has no influence on the tendency of the amplification effect we found, confirming that the original results is robust.
